# Supplementary material for: Neural Networks for Conversion of Simulated NMR Spectra from Low-Field to High-Field for Quantitative Metabolomics
Source: Metabolites. 2024 Dec 1;14(12):666. doi: 10.3390/metabo14120666 (PMC11678772; doi:10.3390/metabo14120666)
Supplement: Supplementary file 1 [file metabolites-14-00666-s001.zip › metabolites-3247490-supplementary.pdf]

Supplementary Data

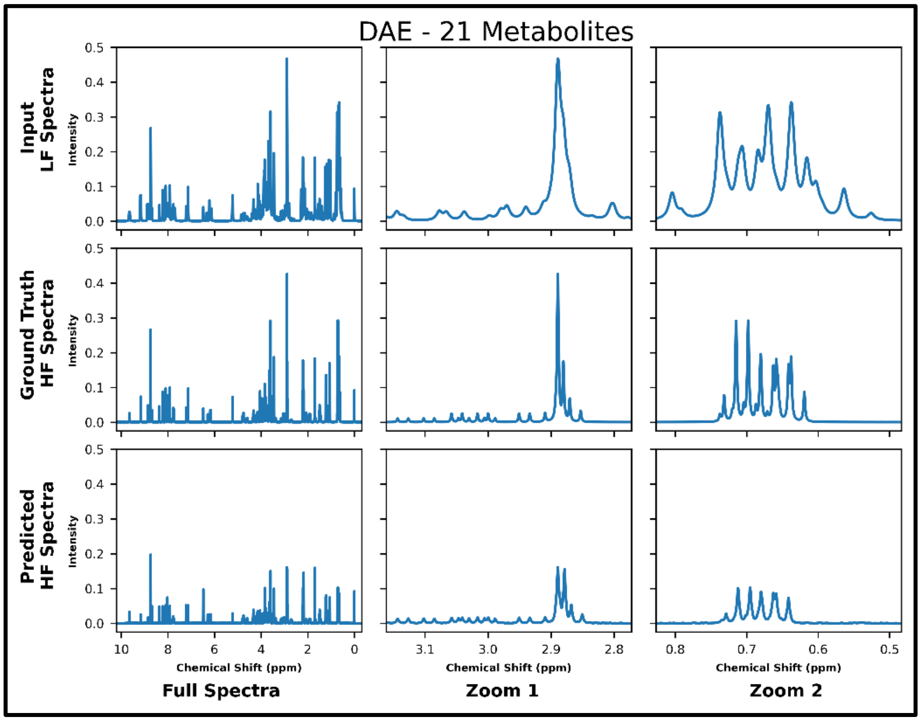

Figure S1. DAE performance results for 100-MHz to 400-MHz conversion of one test spectrum from the dataset of 21 metabolites. The top row shows the input LF spectrum, the middle row shows the ground truth HF spectrum, and the bottom row shows the predicted HF spectrum. The left column shows the full spectra, and the middle and right columns show zoomed in portions of the same spectra. Abbreviations: LF = low-field; HF = high-field; DAE = densely connected autoencoder.

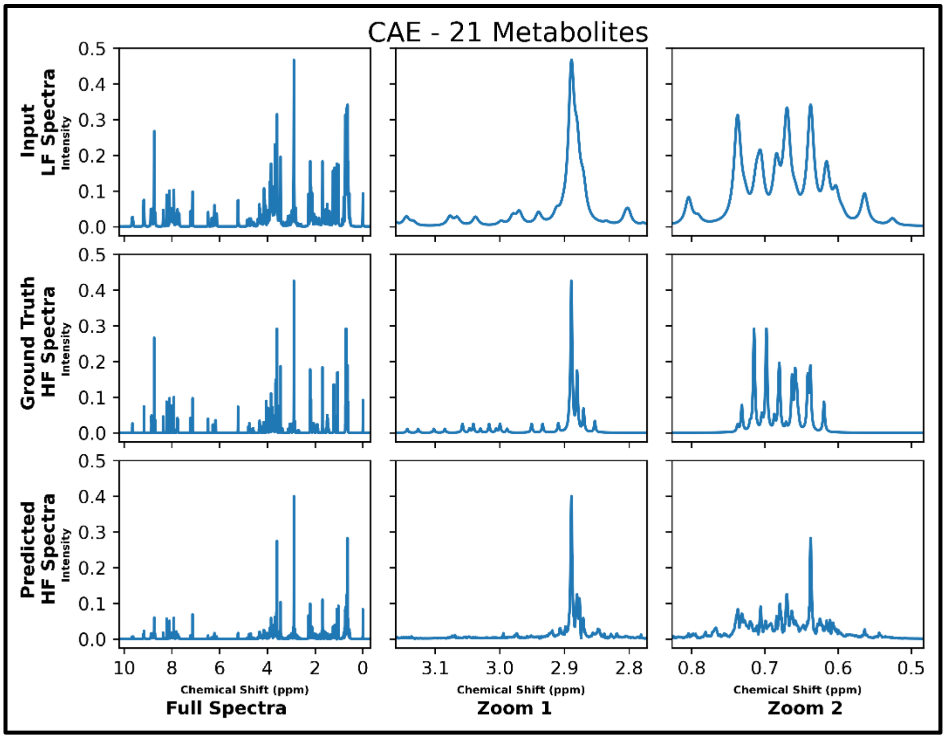

Figure S2. CAE performance results for 100-MHz to 400-MHz conversion of one test spectrum from the dataset of 21 metabolites. The top row shows the input LF spectrum, the middle row shows the ground truth HF spectrum, and the bottom row shows the predicted HF spectrum. The left column shows the full spectra, and the middle and right columns show zoomed in portions of the same spectra. Abbreviations: LF = low-field; HF = high-field; CAE = convolutional autoencoder.

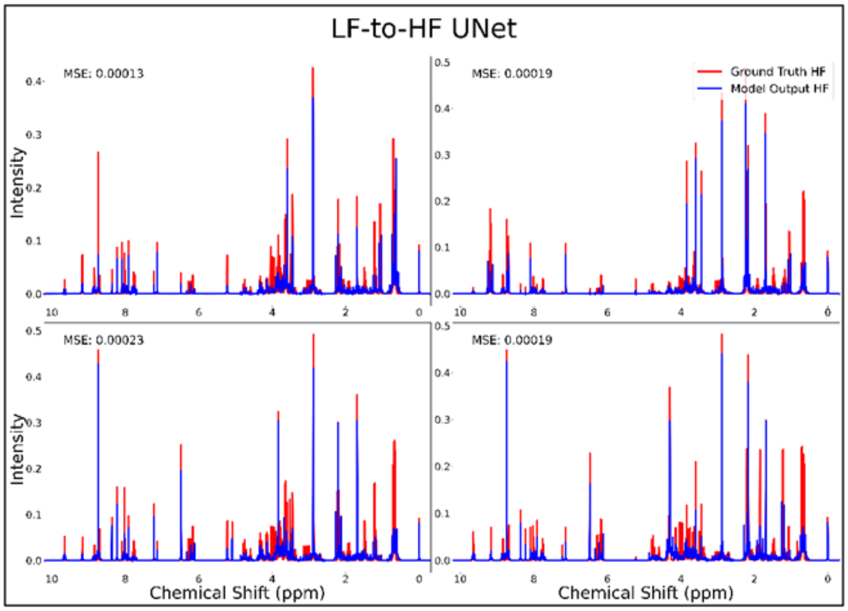

Figure S3. UNet performance results for 100-MHz to 400-MHz conversion of four test spectra from the dataset of 21 metabolites. The ground truth HF spectra is shown in red overlaid with the corresponding predicted HF spectra in blue, and the MSE between spectra is displayed for each conversion. Abbreviations: LF = low-field; HF = high-field; MSE = mean-squared error.

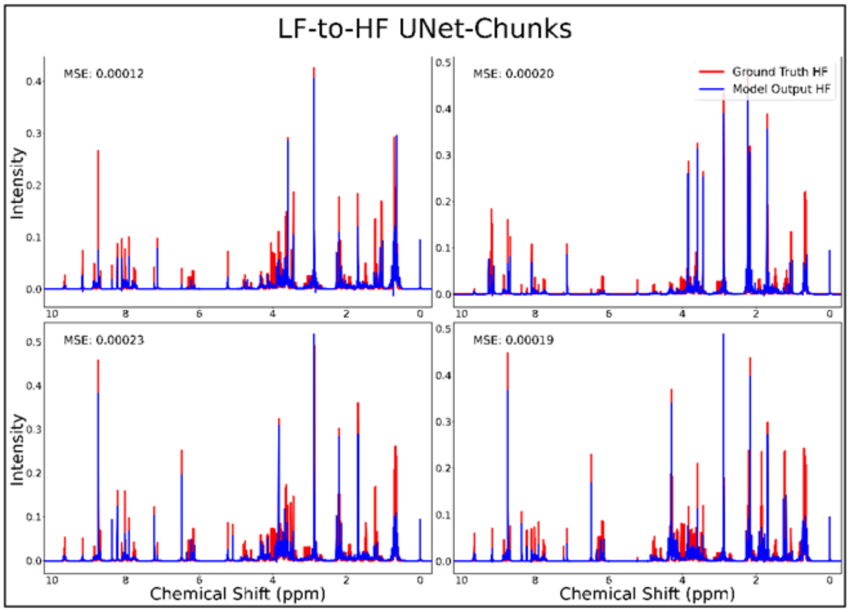

Figure S4. UNet with input separated into bins before inference and concatenated post-inference performance results for 100-MHz to 400-MHz conversion of four test spectra from the dataset of 21 metabolites. The ground truth HF spectra is shown in red overlaid with the corresponding predicted HF spectra in blue, and the MSE between spectra is displayed for each conversion.

Abbreviations: LF = low-field; HF = high-field; MSE = mean-squared error.

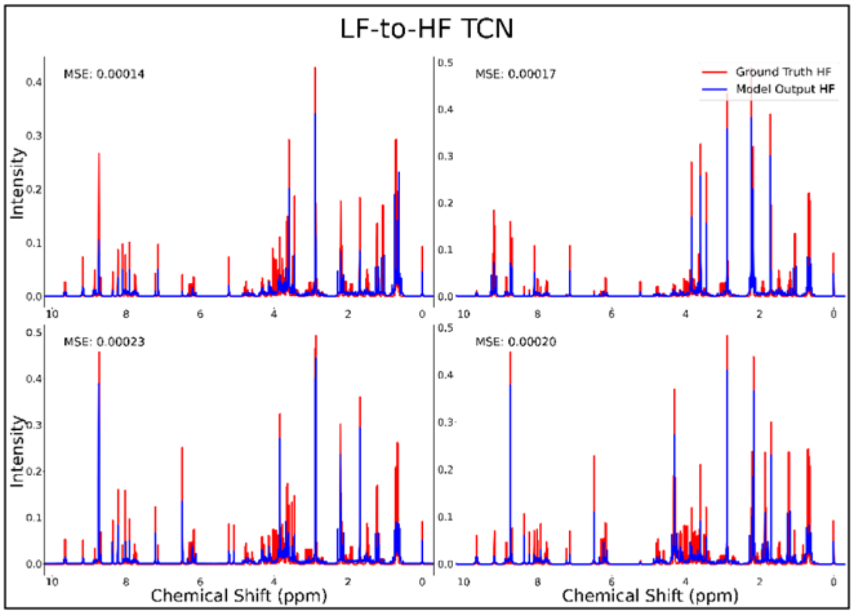

Figure S5. TCN with input separated into bins before inference and concatenated post-inference performance results for 100-MHz to 400-MHz conversion of four test spectra from the dataset of 21 metabolites. The ground truth HF spectra is shown in red overlaid with the corresponding predicted HF spectra in blue, and the MSE between spectra is displayed for each conversion.

Abbreviations: LF = low-field; HF = high-field; TCN = temporal convolutional network; MSE = mean-squared error.

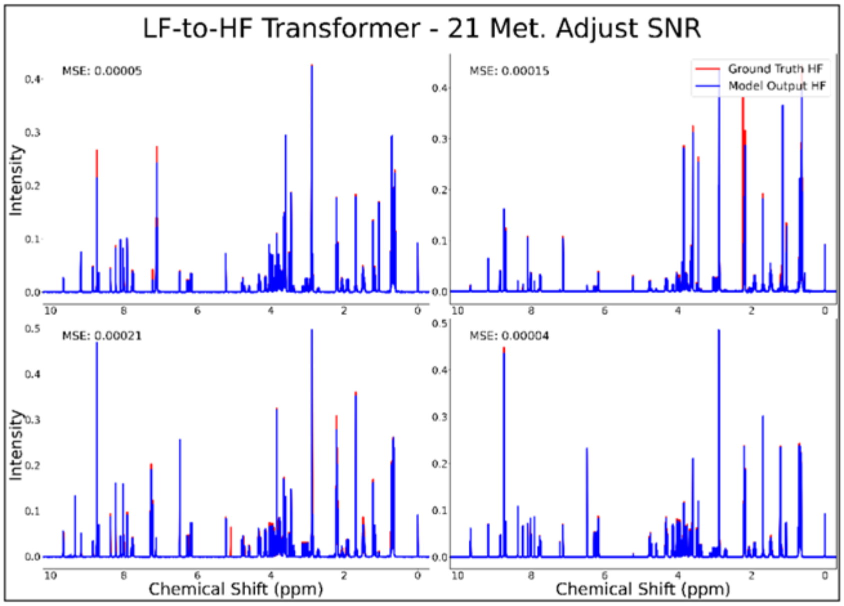

Figure S6. Transformer performance results for 100-MHz to 400-MHz conversion of four test spectra from the dataset of 21 metabolites with adjusted SNR (four times higher noise in 100-MHz compared to 400-MHz spectra). The ground truth HF spectra is shown in red overlaid with the corresponding predicted HF spectra in blue, and the MSE between spectra is displayed for each conversion. Abbreviations: LF = low-field; HF = high-field; SNR = signal-to-noise ratio; MSE = mean-squared error.

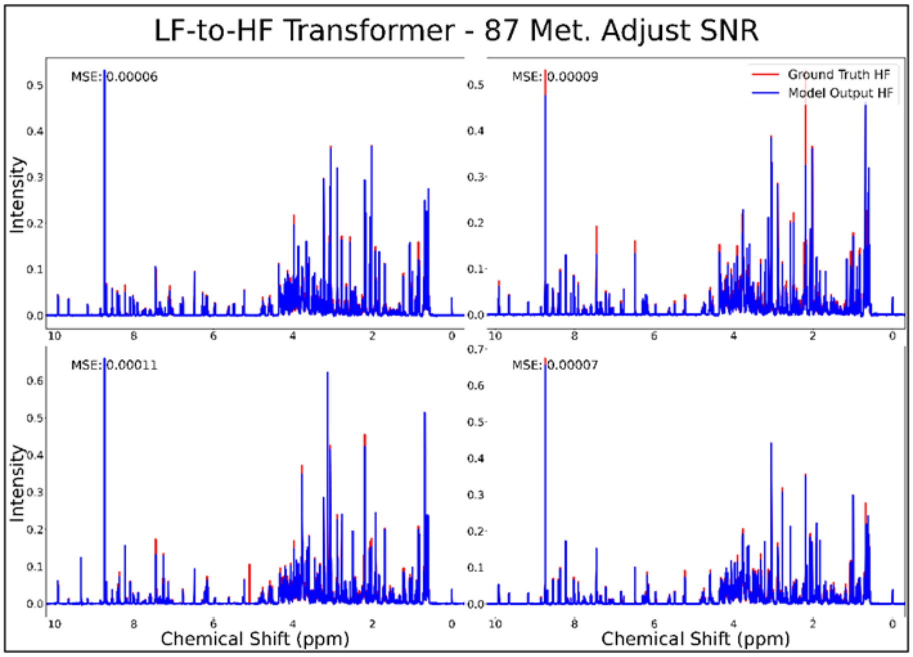

Figure S7. Transformer performance results for 100-MHz to 400-MHz conversion of four test spectra from the dataset of 87 metabolites with adjusted SNR (four times higher noise in 100-MHz compared to 400-MHz spectra). The ground truth HF spectra is shown in red overlaid with the corresponding predicted HF spectra in blue, and the MSE between spectra is displayed for each conversion. Abbreviations: LF = low-field; HF = high-field; SNR = signal-to-noise ratio; MSE = mean-squared error.

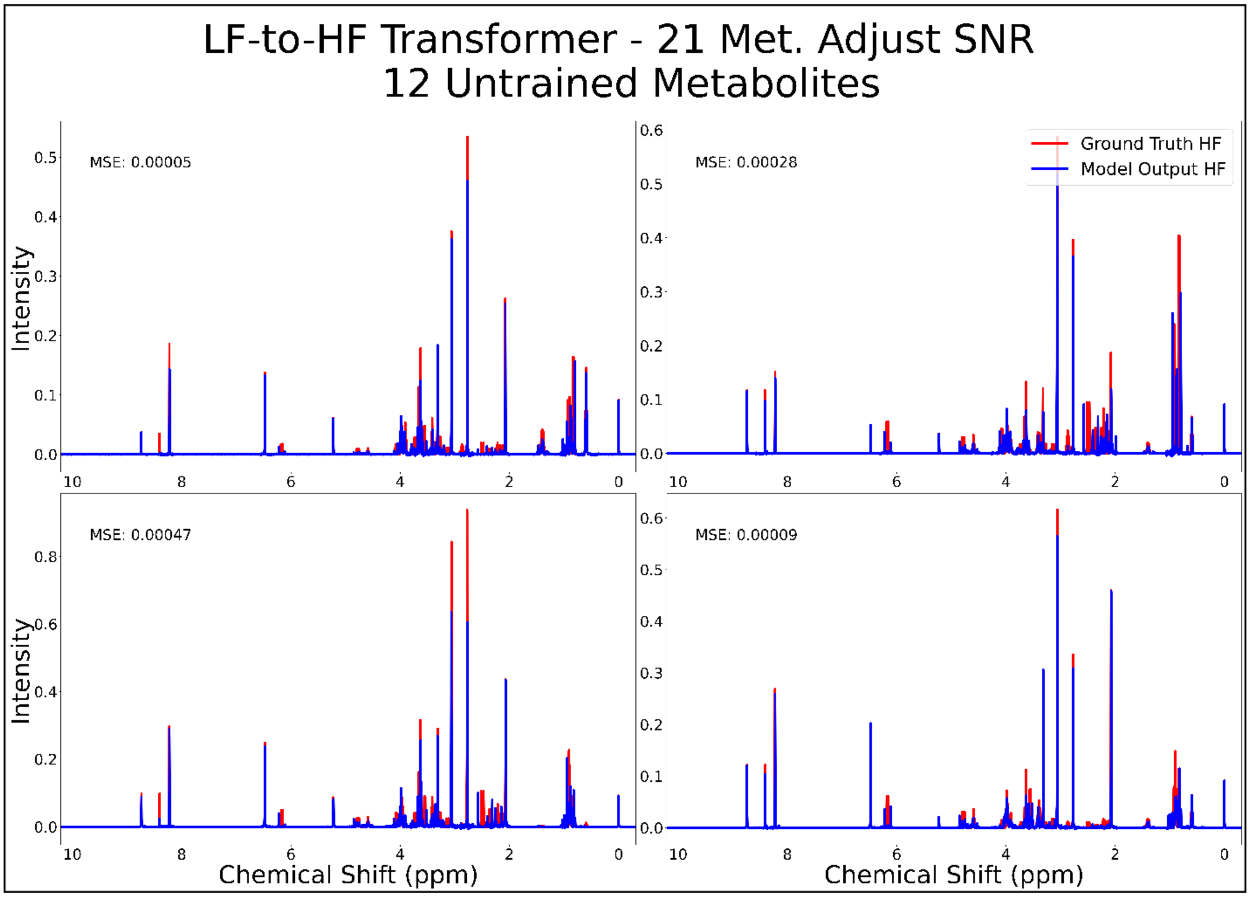

Figure S8. Transformer performance for the model trained on 21 metabolites with adjusted SNR (four times higher noise in 100-MHz compared to 400-MHz spectra) for 100-MHz to 400-MHz conversion of four test spectra from the dataset of 12 metabolites that were not seen by the model in training. The ground truth HF spectra is shown in red overlaid with the corresponding predicted HF spectra in blue, and the MSE between spectra is displayed for each conversion.

Abbreviations: LF = low-field; HF = high-field; SNR = signal-to-noise ratio; MSE = mean-squared error; Met. = Metabolites.

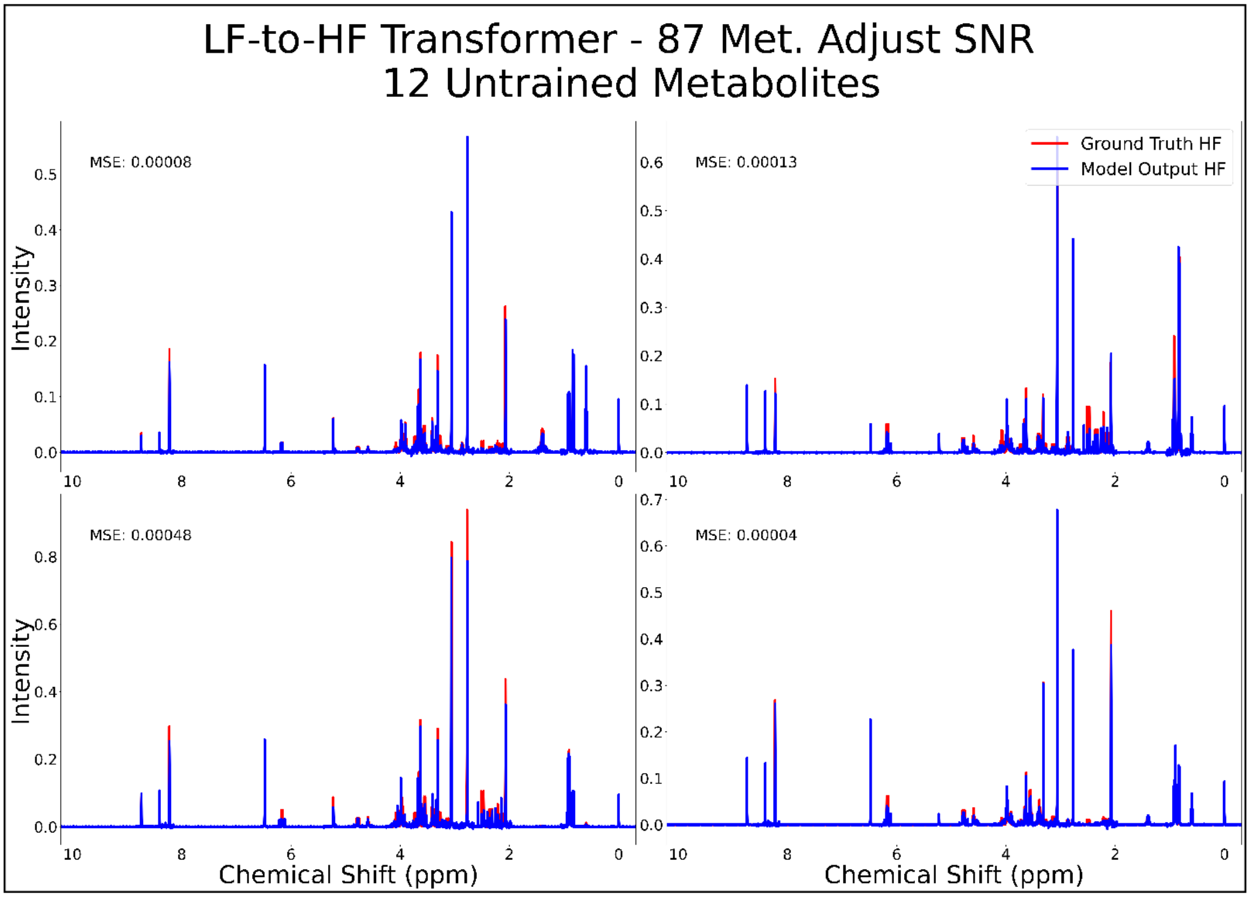

Figure S9. Transformer performance for the model trained on 87 metabolites with adjusted SNR (four times higher noise in 100-MHz compared to 400-MHz spectra) for 100-MHz to 400-MHz conversion of four test spectra from the dataset of 12 metabolites that were not seen by the model in training. The ground truth HF spectra is shown in red overlaid with the corresponding predicted HF spectra in blue, and the MSE between spectra is displayed for each conversion.

Abbreviations: LF = low-field; HF = high-field; SNR = signal-to-noise ratio; MSE = mean-squared error; Met. = Metabolites

		Training Dataset								
		21 Metabolites			21 Metabolites - Adjust SNR			87 Metabolites - Adjust SNR		
		LF	HF	LF-to-HF	LF	HF	LF-to-HF	LF	HF	LF-to-HF
Input Spectra	5 mM	3.9%	6.3%	9.2%	4.0%	6.3%	8.6%	2.7%	3.9%	21%
	25 mM	0.58%	1.1%	1.5%	0.55%	1.1%	1.4%	1.5%	2.9%	6.0%
	50 mM	0.55%	0.84%	1.0%	0.69%	0.84%	1.2%	1.5%	2.9%	8.5%

Table S1. Performance for direct LF spectra quantification using the LF-MLP, direct HF quantification using the HF-MLP, and LF-to-HF conversion prior to quantification using the HF-MLP. Percentages shown are the mean absolute percent error (MAPE) for each model on three testing spectra containing 21 (or 87 depending on the dataset) metabolites all at 5, 25, or 50 mM.

Abbreviations: HF = high-field; LF = low-field; SNR = signal-to-noise ratio.
